# Validation of remote anthropometric measurements in a rural randomized pediatric clinical trial in primary care settings

**DOI:** 10.1038/s41598-023-50790-1

**Published:** 2024-01-03

**Authors:** E Zhang, Ann M. Davis, Elizabeth Yakes Jimenez, Brittany Lancaster, Monica Serrano-Gonzalez, Di Chang, Jeannette Lee, Jin-Shei Lai, Lee Pyles, Timothy VanWagoner, Paul Darden

**Affiliations:** 1 Department of Occupational Therapy Education, University of Kansas Medical Center, 3901 Rainbow Blvd, Kansas City, KS 66160 USA; 2 Department of Pediatrics, University of Kansas Medical Center, Kansas City, KS USA; 3 College of Population Health and Departments of Pediatrics and Internal Medicine, University of New Mexico Health Sciences Center, Albuquerque, NM USA; 4 Department of Pediatrics, Warren Alpert Medical School of Brown University, Providence, RI USA; 5 Department of Biostatistics, University of Arkansas for Medical Sciences, Little Rock, AR USA; 6 Department of Medical Social Sciences, Northwestern University Feinberg School of Medicine, Chicago, IL USA; 7 Department of Pediatrics, West Virginia University, Morgantown, WV USA; 8 Department of Pediatrics, University of Oklahoma Health Sciences Center, Oklahoma City, OK USA; 9 Department of Pediatrics, University of Arkansas for Medical Sciences, Little Rock, AR USA

**Keywords:** Paediatric research, Weight management

## Abstract

Rural children are more at risk for childhood obesity but may have difficulty participating in pediatric weight management clinical trials if in-person visits are required. Remote assessment of height and weight observed via videoconferencing may provide a solution by improving the accuracy of self-reported data. This study aims to validate a low-cost, scalable video-assisted protocol for remote height and weight measurements in children and caregivers. Families were provided with low-cost digital scales and tape measures and a standardized protocol for remote measurements. Thirty-three caregiver and child (6–11 years old) dyads completed remote (at home) height and weight measurements while being observed by research staff via videoconferencing, as well as in-person measurements with research staff. We compared the overall and absolute mean differences in child and caregiver weight, height, body mass index (BMI), and child BMI adjusted Z-score (BMIaz) between remote and in-person measurements using paired samples t-tests and one sample t-tests, respectively. Bland–Altman plots were used to estimate the limits of agreement (LOA) and assess systematic bias. Simple regression models were used to examine associations between measurement discrepancies and sociodemographic factors and number of days between measurements. Overall mean differences in child and caregiver weight, height, BMI, and child BMIaz were not significantly different between remote and in-person measurements. LOAs were − 2.1 and 1.7 kg for child weight, − 5.2 and 4.0 cm for child height, − 1.5 and 1.7 kg/m^2^ for child BMI, − 0.4 and 0.5 SD for child BMIaz, − 3.0 and 2.8 kg for caregiver weight, − 2.9 and 3.9 cm for caregiver height, and − 2.1 and 1.6 kg/m^2^ for caregiver BMI. Absolute mean differences were significantly different between the two approaches for all measurements. Child and caregiver age were each significantly associated with differences between remote and in-person caregiver height measurements; there were no significant associations with other measurement discrepancies. Remotely observed weight and height measurements using non-research grade equipment may be a feasible and valid approach for pediatric clinical trials in rural communities. However, researchers should carefully evaluate their measurement precision requirements and intervention effect size to determine whether remote height and weight measurements suit their studies.

Trial registration: ClinicalTrials.gov NCT04142034 (29/10/2019).

## Introduction

The prevalence of pediatric obesity in the U.S. remains a critical public health concern. The Centers for Disease Control and Prevention data (2017–2018) indicate that 19.3% of US children and adolescents (ages 2–19 years old) are classified as obese (BMI ≥ 95th percentile) and another 16.1% as overweight (BMI ≥ 85th percentile)^1^. This is a serious issue, as childhood obesity has many short-term and long-term health consequences^2–4^. Children living in rural areas are more likely to be overweight and obese when compared to their urban peers^5^. Obesity treatment and preventative interventions commonly involve anthropometric measurements such as height and weight to assess eligibility and progress during treatment. For research studies, anthropometric measurement protocols typically involve trained staff measuring children using research-grade equipment during in-person appointments. However, in-person measurements can pose logistical challenges when conducting studies in remote and rural areas and when in-person measurements are not allowed, such as during the early phases of the COVID-19 pandemic.

To meet the need for alternative measurement options, some researchers have examined the feasibility and validity of having participants complete and self-report remote height and weight measurements^6,7^. These methods have proven feasible, but their validity is questionable due to the increased potential for biases or errors in measurement^6^. This is particularly true for studies involving children. In these cases, caregivers are typically responsible for measuring their child’s height and weight, which may introduce additional errors due to bias or lack of knowledge regarding best practices, or because children can be particularly difficult to measure^8^.

Findings from studies conducted thus far comparing anthropometric measurements of school-age children performed independently by caregivers at home with measurements taken by professionals in school or clinical settings, have been mixed, depending on the measurement considered. Tenenbaum et al.^9^ found that heights measured by parents at home were comparable to those taken by clinic staff using clinic equipment, except in overweight and obese children whose heights were significantly higher in the clinic. Weight measured at home and the calculated BMI were significantly lower than those measured in the clinic for all youth. Sarkkola et al.^10^ also assessed the validity of home-measured height, weight, and waist circumference among Finnish youth (9–11 years old). They compared measurements taken by parents using a measuring tape provided by the study team and home scales with measurements conducted using a calibrated portable stadiometer and digital scales by trained assessors at school. Results showed that home-measured height, weight, and waist circumference were higher than at school. The difference in means was significant for the weight (0.51 kg) and waist circumference (1.6 cm) but not for height and BMI. Forseth et al.^11^ examined the feasibility and validity of home-measured heights and weights with rural school-age youth and their caregivers in a school-based healthy lifestyle intervention trial. The overall difference in means was not statistically significant for the weight (0.10 kg) or height (0.26 cm) in children but was approaching statistical significance in caregivers’ weight (0.22 kg) when comparing home measurements taken using inexpensive scales and tape measures with measurements conducted by the study team using research-grade equipment. However, Forseth et al.^11^ identified statistically significant differences in child and caregiver weight, child height, child BMI percentile, and caregiver BMI using the absolute mean difference statistic. This study and others cited above relied on caregiver self-report, which limits any possibility of examining caregiver adherence to the measurement protocol.

To increase the validity of remote measurements, Ghosh et al.^12^ explored using videoconferencing to observe and guide caregivers while they completed the remote measurements, as a potential improvement over relying on self-report alone. They examined the validity of videoconferencing observed height, weight, percent body fat, and waist circumference measurements relative to in-person measurements by trained staff using the same equipment with a convenience sample of 10 parent–child dyads and 15 adult-adult dyads either in participants’ homes or the researcher’s office. The height was measured using a Charder HM200P Portstad Portable Stadiometer ($115), and the weight was measured by a Tanita UM-081 Scale ($64.99). The small magnitude of differences (effect sizes < 0.03) and high agreement suggest that adding videoconferencing to observe the height and weight measurements may increase the validity of the remote measurement approach.

Although the findings of the study using the observed videoconferencing protocol^12^ are promising, the protocol for that study may not be widely scalable and fiscally practical in large clinical trials or cohort studies. Particularly, at a budget of about $180 per participant, using this remote measurement protocol with large samples of caregivers and children may prove challenging. Therefore, the current study aims to validate an affordable, widely scalable video-assisted protocol for obtaining height and weight remotely in caregivers and their children. We also explore factors that might cause the discordance between in-person and remote measurement.

## Methods

### Participants

The study participants were part of the Feasibility Trial of the iAmHealthy Intervention for Healthy Weight in Rural Children Recruited from Primary Care Clinics (iAmHealthy; ClinicalTrials.gov ref. NCT04142034, 29/10/2019) through Environmental Influences on Child Health Outcomes IDeA States Pediatric Clinical Trials Network (ECHO ISPCTN) sites. Families of children attending four rural primary care clinics who met the following inclusion criteria were invited to participate (one child and one caregiver): ages 6–11 years old, BMI percentile ≥ 85th, household located in a rural area, and child and primary caregiver spoke English. Rurality was defined as United States Department of Agriculture Rural–Urban Commuting Area (RUCA) codes greater than or equal to 4^13^. Detailed study methods are reported elsewhere^14,15^.

### Procedure

The iAmHealthy feasibility trial was initiated in early 2020 with four ISPCTN sites in Nebraska, South Carolina, Delaware, and West Virginia to evaluate feasibility related to participant recruitment, participant retention, intervention dose, and blinding to prepare for a larger randomized controlled trial^16^. The recruitment effort was initiated in February 2020 and was halted on March 13, 2020, due to the onset of the COVID-19 pandemic in the U.S.^17^. The study team amended the study protocol to adapt to pandemic-related research restrictions limiting in-person research contact at participating sites. The changes included switching key outcome measures from in-person to remote collection, including height and weight measurements. The study team followed CDC guidance^18^ and prior research on measuring height and weight at home^8,12^ and used a detailed protocol.^11^ (see Table 1). The protocol was provided to both caregivers and the study personnel who observed the measurement session and completed in-person measurements. The study team from each site shipped equipment, including a digital scale, measuring tape, painter’s tape, and ruler, to each participating family.

**Table 1:** Protocol for standardization of measuring height and weight at home through videoconferencing

**Instructions for research staff**
Caregivers and children will measure their height and weight at home with the help of an iAmHealthy study team member. The families will measure their height and weight on a video call with an iAmHealthy study team member. The team member will give the family instructions on how to obtain height/weight and will observe the measurement over the video call to ensure accuracy. The families will report the height and weight to the team member during the video call, and the team member will log it in the study records
Instructions for caregivers
**Steps to measure your child’s height**
1. First, find a flat, uncarpeted section of the floor and a flat section of the wall 2. Have your child remove their shoes 3. Remove braids, headbands, or anything else on your head that may get in the way of an accurate measurement 4. Remove any bulky clothing that may make it difficult to stand flat against the wall 5. Have your child stand with their feet flat on the floor with their heels against the corner where the wall and floor meet. Make sure their head, shoulders, and buttocks are touching the wall 6. Have your child stand up straight with their eyes looking straight ahead. Your child’s chin should be parallel to the floor 7. Place a flat object (like a rule or hardcover book) against the wall at a right angle. Then lower it until it rests gently on top of your child’s head, keeping it at a right angle to the wall 8. Lightly mark the wall (or a piece of masking tape that you have adhered to the wall) with a pencil at the point the ruler or book (or other flat objects) meets your child’s head 9. Use a tape measure to measure the distance from the floor to the mark on the wall 10. Take note of the measurement to the nearest 1/8^th^ of an inch or 0.1 cm
Steps to measure you and your child’s weight 1. Place a scale on a flat, uncarpeted floor section 2. Remove your/your child’s shoes and any heavy objects (jackets, sweatshirts, coats, etc.). Remove items from your pockets (wallet, keys, etc.) 3. If the scale is digital, tap with your foot so the scale turns on. Remove your foot
1. Once the scale displays “0.0”, have you or your child step on the scale 2. Hold still for several seconds until the number on the scale stops moving 3. Take note of the measure to the nearest 0.01 lb

At the post-intervention measurement, two of the four clinics (South Carolina and Delaware) lifted their in-person visit restrictions and allowed participants to have data collected both remotely and in-person, which presented an opportunity for validating the caregiver protocol to measure remote height and weight. All measures for this validation study across both sites were completed between February 8 and March 7, 2021. These protocol changes were approved by the Data Safety and Monitoring Board (DSMB), the National Institutes of Health (NIH), and the University of Arkansas for Medical Sciences Institutional Review Board (central IRB for the study). Written informed consent approved by the central IRB (cIRB #: 249932) and/or child assent were obtained for all participants.

Blinded ECHO ISPCTN site assessors completed training for implementing both remote and in-person height and weight measurements. They measured the height and weight of three people at their site following the protocol in Table 1 and reached inter-rater reliability, with less than a 5% difference with a backup-blinded assessor. The site coordinators assessed the clinic stadiometer and scale monthly for reliability and calibrated the equipment before each measurement. Blinded assessors were instructed to complete remote and in‐person measurements as close together as possible, in any order.

#### Remote measurement visits via videoconferencing

The blinded assessors guided caregivers through remote measurements using HIPAA-compliant Zoom (Zoom Video Communications, Inc., San Jose, CA) to observe the measurement and verbally obtain and record the child’s height and weight and caregiver height and weight measurements in triplicate. Caregivers were provided with the measurement protocol in advance of the videoconferencing visit.

#### In-person measurement visits

The blinded assessors obtained the height and weight measurements in triplicate for both the caregiver and child participants in the two primary care clinics. The measurements were conducted following the same protocol (Table 1).

### Measures

#### Weight

Home scale weights were taken using standardized equipment (Etekcity High Precision Digital Body Weight Bathroom Scale with Ultra-Wide Platform and Easy-to-Read Backlit LCD; model number 025706343039; 440 lb; $26.99), accurate within 0.2 lb. Clinic weights were measured using the SECA Model 813 portable digital scale (SECA, Hamburg, Germany; 440 pounds; $102), accurate within 0.2 lb over a range from 1 to 440 lb. All weights were measured in triplicate, with participants wearing light clothing and no shoes, and were recorded to the nearest 0.01 lb (for home weights) or 0.1 kg (for clinic weights). Mean values were used in the analyses.

#### Height

Child home height was measured by their caregiver using a tape measure provided by the research team (Amazon Basics Tape Measure, model number DS-TAM10-16ft; $9). Child clinic height was measured on a Detecto Free‐Standing Portable Height Rod (4.5 in–81 in/11.5 cm–205 cm: $165). During height measurements, participants were instructed to remove their shoes, stand against the wall, and look straight ahead, following the detailed procedures outlined in Table 1. All heights were taken in triplicate and recorded to the nearest 0.1 cm; the mean of the three measures was used in analyses. The caregiver's height was measured at home in the same manner by another adult following the procedure in Table 1. The caregiver’s self-reported height was recorded when another adult was unavailable.

#### Demographic information

Caregivers completed the iAmHealthy Demographics Form at baseline, which contained questions about child and caregiver age, sex, race, ethnicity, household income, insurance status, caregiver education, and zip code (used to determine rurality using RUCA codes).

### Data processing

We calculated BMI in kg/m^2^ for children and caregivers using the height and weight data collected remotely and in-person. Researchers also calculated the BMI adjusted Z-score (BMIaz) for children to measure childhood adiposity change using methods outlined by Freedman & Berenson^19^. BMIaz is calculated as the BMI z-score adjusted to the 95th percentile z-score value for age and gender. BMIaz is designed to be more sensitive to change for children with BMI percentiles over the 97th percentile, especially for those under 10 years of age^19^. Similarly, BMI as a percent of 95th percentile value can be employed, and any BMI greater than 120% of the 95%ile is considered severe obesity.

We then calculated the absolute mean difference (i.e., average of the absolute values of the differences between remote and in-person measurements) and the overall mean difference (i.e., average differences between remote and in-person measurements) for child and caregiver height, weight, BMI, and child BMIaz. As pointed out by Forseth et al.^11^, the analysis of the absolute mean difference (i.e., the average magnitude of differences regardless of their direction) can help us understand the impact of measurement discrepancies on individual-level outcomes.

### Data analysis

Descriptive statistics were calculated for participant sociodemographic characteristics. The mean, standard deviation, and 95% confidence interval (CI) for remote and in-person child and caregiver height, weight, BMI, and child BMIaz were also calculated. All data analyses were carried out with version 9.4 of the SAS System for Windows (SAS Institute Inc., Cary, NC, USA).

#### Absolute mean difference

We used one-sample t-tests to determine if the mean absolute value of the difference between measurements was significantly different from 0. The mean, standard deviation, and 95% CI of the absolute mean difference between approaches for child and caregiver weight, height, and BMI and child BMIaz are reported.

#### Overall mean difference

We used paired samples t-tests to examine within-person differences between remote and in-person child and caregiver weight, height, BMI, and child BMIaz to determine if there were systematic differences, i.e., one measure was consistently higher than the other. The average within-person difference between approaches, its standard deviation, and the 95% CI are reported. The agreement between the remote and in-person measurements was further investigated by examining Bland–Altman plots^20^. The Bland–Altman plots were used to estimate the limits of agreement [LOA], or the interval within which 95% of the differences between the two measurements fall, and to examine whether the differences between remote versus in-person measurements were consistent across the range of the average values of child and caregiver weight, height, and BMI and child BMIaz measures. Additionally, we computed intraclass correlation coefficients (ICCs) to assess how variability in child and parent height and weight measurements could be attributed to the differences between remote and in-person assessment methods.

#### Exploratory analysis

Simple linear regression was used to individually examine whether child age, sex, caregiver age and education level, or the number of days between measures were associated with the measurement discrepancies in child and caregiver weight, height, BMI, and child BMIaz.

### Ethics approval and consent to participate

All study procedures were approved by the Institutional Review Board at the University of Arkansas for Medical Sciences through the SMART IRB system, with all other sites relying on UAMS as the central IRB. Participants provided informed consent prior to data collection. Statement of human subjects: All methods were carried out in accordance with relevant guidelines and regulations, and all participants provided Informed consent and/or assent prior to their involvement in the study. Informed consent was provided by all the parents or legal guardians of the minors who were involved in this study.

## Results

### Sample characteristics

Height and weight were measured for 40 child and caregiver dyads remotely and in- person. However, five pairs of measurements were excluded because the number of days between the two measures was greater than seven days (mean = 22, SD = 11.8). In addition, measurements for two pairs were deemed outliers because there were large, biologically implausible differences between remote and in-person measurements. One caregiver’s weight differed by 22.4 lb. (− 1.68 days between two measurements), and one child’s height differed by 22.0 cm (− 3.75 days between two measurements). In both cases, the remote measurements were lower compared to in-person measurements.

Therefore, 33 child and caregiver dyads were included in this analysis. Their sociodemographic characteristics are presented in Table 2. The children had a mean age of 9.3 ± 1.7 (SD) years, and 60.6% were female. The caregivers had a mean age of 38.8 ± 10.7 (SD) years, and 93.9% of them were female. Most participants were African American or White. About two-thirds of caregivers did not have a college degree. The average time between the remote and in-person measurements for the 33 dyads was − 2.2 ± 3.3 (SD) days.

**Table 2:** Sociodemographic characteristics of children and caregivers.

Child characteristics (n = 33)	
Age (years), mean (standard deviation [SD]), median, min, max	9.3 (1.7), 9.5, 6.6–11.9
Sex (female), n (%)	20 (60.6)
Ethnicity (Hispanic or Latino), n (%)	4 (12.1)
Race, n (%)	
Black or African American	15 (45.5)
White	14 (42.4)
Multiracial	3 (9.1)
Puerto Rican	1 (3.0)

Caregiver characteristics (n = 33)	
Age (years), mean (SD), median, min, max	38.8 (10.7), 37, 26.0–81.0
Sex (female), n (%)	31 (93.9)
Ethnicity (Hispanic or Latino), n (%)	4 (12.1)
Race, n (%)	
Black or African American	16 (48.5)
White	14 (42.4)
Multiracial	2 (6.1)
Puerto Rican	1 (3.0)
Education level, n (%)	
College degree	11 (33.3)
No college degree	22 (66.7)
Marital Status, n (%)	
Married	14 (42.4)
Single/never married	14 (42.4)
Missing	5 (15.2)
Household Income, n (%)	
< = $39,999	21 (63.6)
$40,000–$79,999	7 (21.2)
> = $80,000	5 (15.2)

### Anthropometric differences

The overall mean differences and absolute mean differences for child and caregiver weight, height, and BMI and child BMIaz are presented in Table 3. The overall mean differences were not significantly different for any of the child or caregiver measures. However, the absolute mean differences were significantly different from zero for all child and caregiver measures.

**Table 3:** Descriptive comparison of remote and in-person measurements (n = 33 child and caregiver dyads)

	Remote measurement (at home)	In-person measurement (at clinic)	Overall mean difference^a^			Absolute mean difference^a^		
Weight (kg)				p-value^b^	d		p-value^c^	d
Child Mean (SD) 95% CI	55.5 (13.6) (50.7, 60.3)	55.7 (13.4) (50.9, 60.4)	− 0.2 (1.0) (− 0.5, 0.1)	0.260	− 0.01	0.7 (0.7) (0.4, 0.9)	< 0.001	0.89
Caregiver Mean (SD) 95% CI	95.4 (24.4) (86.7, 104.0)	95.5 (24.4) (86.8, 104.1)	− 0.1 (1.5) (− 0.6, 0.4)	0.703	0.00	1.1 (1.0) (0.7, 1.4)	< 0.001	1.10
Height (cm)								
Child Mean (SD) 95% CI	146.4 (10.9) (142.5, 150.2)	147.0 (10.7) (143.2, 150.8)	− 0.6 (2.3) (− 1.4, 0.2)	0.150	− 0.06	1.7 (1.7) (1.1, 2.3)	< 0.001	0.98
Caregiver Mean (SD) 95% CI	165.5(8.2) (162.6, 168.4)	165.0 (8.2) (162.1, 167.9)	0.5 (1.7) (− 0.1, 1.1)	0.113	0.06	1.2 (1.3) (0.8, 1.7)	< 0.001	0.94
BMI								
Child Mean (SD) 95% CI	25.6 (4.0) (24.2, 27.0)	25.5 (4.1) (24.0, 26.9)	0.1 (0.8) (− 0.2, 0.4)	0.487	0.03	0.6 (0.6) (0.4, 0.8)	< 0.001	1.11
Caregiver Mean (SD) 95% CI	34.7 (7.9) (31.9, 37.5)	34.9 (7.9) (32.1, 37.7)	− 0.2 (1.0) (− 0.6, 0.1)	0.152	− 0.03	0.7 (0.6) (0.5, 1.0)	< 0.001	1.15
BMIaz^d^								
Child Mean (SD) 95% CI	2.3 (1.0) (1.9, 2.6)	2.3 (1.1) (1.8, 2.6)	0.0 (0.2) (− 0.0, 0.1)	0.402	0.03	0.1 (0.2) (0.1, 0.2)	< 0.001	0.94

^a^Difference = remote-in-person measurement value.

^b^Paired sample t-tests.

^c^One-sample t-tests.

^d^BMIaz is BMI considered as a z-score adjusted to 95th percentile BMI for age and gender.

**Figure 1:**
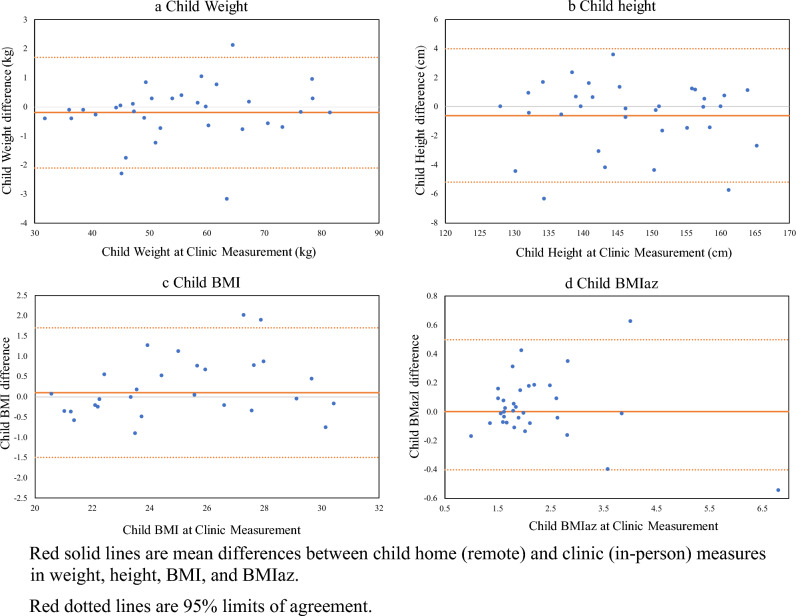
Bland–Altman plots for child remote (at home) and in person (at clinic) measurements in weight, height, BMI, and BMIaz. Red solid lines are mean differences between child home (remote) and clinic (in-person) measures in weight, height, BMI, and BMIaz. Red dotted lines are 95% limits of agreement.

**Figure 2:**
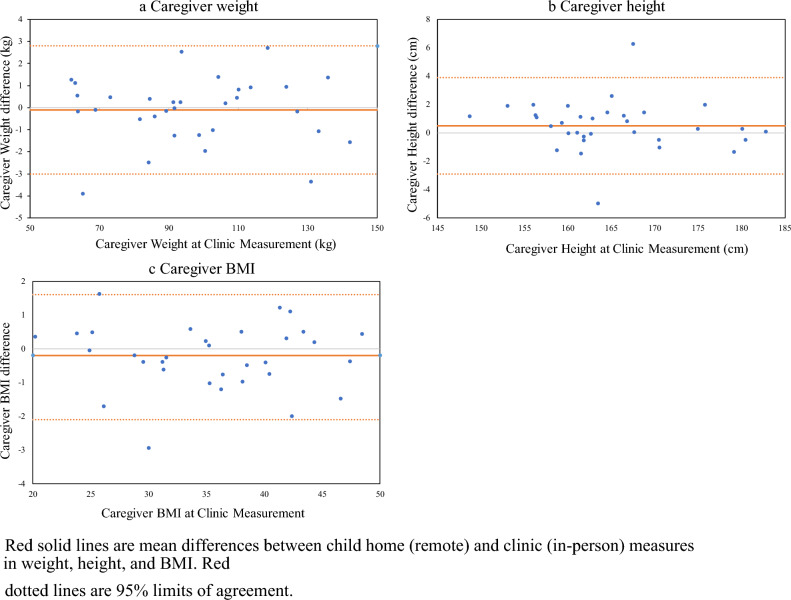
Bland–Altman plots for caregiver remote (at home) and in person (at clinic) measurements in weight, height, and BMI. Red solid lines are mean differences between child home (remote) and clinic (in-person) measures in weight, height, and BMI. Red dotted lines are 95% limits of agreement.

The LOA indicated by the Bland–Altman plots were − 2.1 and 1.7 kg for child weight (Fig. 1a), − 5.2 and 4.0 cm for child height (Fig. 1b), − 1.5 and 1.7 kg/m^2^ for child BMI (Fig. 1c), and − 0.4 and 0.5 SD for child BMIaz (Fig. 1d). For caregivers, the LOA were − 3.0 and 2.8 kg for weight (Fig. 2a), − 2.9 and 3.9 cm for height (Fig. 2b), and − 2.1 and 1.6 kg/m^2^ for BMI (Fig. 2c). Visually, the patterns of measurement differences did not change across the average values of the weight, height, BMI, and BMIaz measures. Most of the values fell within the LOAs, without systematic bias detected. The ICC for child weight is 0.999, 95% CI [0.997, 0.999], and the ICC for child height is 0.988, 95% CI [0.976, 0.994]. For caregivers, the ICC is 0.999, 95% CI [0.998, 1.000] for weight and 0.989, 95% CI [0.977, 0.994] for height.

### Factors associated with discrepancies in measurements

In simple regression models, child age and caregiver age, when categorized as younger or older than their respective median ages, were each significantly associated with differences between remote and in-person caregiver height measurements, β = − 1.4, p < 0.05, and β = − 1.3, p < 0.05. Children younger than the median age of 9.5 years and caregivers younger than 37 years are each associated with a decreased caregivers’ height difference than those older than the median ages. For all other anthropometric measurements, there were no significant associations between child and caregiver sociodemographic variables or time between measurements and measurement discrepancies in simple regression models (Data are not included).

## Discussion

In this study, we assessed the validity of height and weight measurements obtained by caregivers following an affordable, widely scalable, remote video-assisted at-home protocol compared with measurements obtained by trained assessors using research-grade equipment in-person at a clinic. We found that overall mean differences in child and caregiver weight, height, BMI, and child BMIaz were small and not significantly different between remote and in-person measurements. However, LOAs potentially spanned clinically meaningful differences, and absolute mean differences were significantly different between the two approaches for all measurements and potentially clinically meaningful. For example, the absolute mean difference in child height was 1.7 cm, which is equivalent to about 1/3 of the expected yearly growth for a school-age child before puberty^21^. The discrepancy of absolute vs overall mean difference may indicate that the remote method can be used to assess population but not individual differences. The child and caregiver age, as categorized by younger or older than their respective medians, were significantly associated with differences between remote and in-person caregiver height measurements. Additional quality control measures may be needed for remote height measurements for older children and caregivers.

Our findings are generally consistent with those of Ghosh et al.^12^, who used remote observation but more expensive remote equipment, and Forseth et al.^11^, who used a similar measurement protocol, with a similar population (8.3 ± 0.7 years; 66% female) and remote equipment but did not include remote observation. However, there were more discrepancies in the absolute mean difference compared to Forseth et al.^11^ and overall mean difference in weight, height, and BMI measures in our study compared to Ghosh et al.^12^ and Forseth et al.^11^. One factor that may explain this difference is that the number of days between the remote and in-person measurements was greater in this study compared with those studies, although the number of days between measurements was not significantly associated with measurement discrepancies in the current study. In the Ghosh et al.^12^ study, remote and in-person measurements were conducted in the same session. In the Forseth et al.^11^ study, all but one child participant was weighed on the same day at school and at home. Other possible factors include the variability of when the measurements were conducted, and the precision of the equipment used at home. Specifically, body weight fluctuates throughout the day^22^, and remote measurements in the current study were done at various times; equipment used at home was not calibrated because it was shipped directly to the home due to COVID-19 restrictions.

In contrast to findings from some other studies that compared self-report (without videoconferencing) and research staff-measured anthropometric data in both adults^7,23^ and children^9^, our study had smaller 95% LOAs for child BMI and BMIaz and caregiver BMI. However, the LOAs in our study are larger than the LOAs in Forseth et al.^11^ and Ghosh et al.^12^. Smaller LOAs are advantageous in that measurement discrepancies would be less likely to completely span or exceed the anticipated impact for weight management interventions at the individual level. The LOAs observed in this study could span clinically meaningful ranges of impact. For example, in this study, the LOA for child BMIaz was − 0.4 and 0.5; depending upon the methods of the study, a difference of this size may or may not have an impact on study findings.

Researchers can consider implementing remote measurements with both scientific and logistical issues in mind. Although the difference at the group level was not significant, measurement type may have a considerable impact at the individual level. Overall, the results suggest that remote anthropometric measurements are acceptable to use at the group level and will not have a systematic impact on the interpretation of clinical trial results involving child BMIaz or adult BMI. However, researchers should carefully evaluate their measurement precision requirements and intervention effect size to determine whether remote height and weight measurements are suitable for their studies. Logistically, factors such as limited availability of reliable internet in the study area, potentially limited privacy in participants’ homes, and limited participant digital literacy can influence feasibility. Although our study did not formally collect specific data on logistical problems during the remote measurement sessions, staff did not report issues that impeded the remote data collection. A detailed measurement protocol and training for remote data collection were key to success.

Our study used videoconferencing-assisted remote measurement of weight and height in participants’ homes using non-research grade equipment. The potential implications of this approach for future research on obesity interventions are significant. Researchers can potentially collect data from geographically dispersed samples without the expensive deployment of trained personnel and research-grade equipment. This approach may benefit underserved and rural populations with limited access to healthcare facilities, transportation, or trained personnel. It could potentially be adapted and validated in the future to collect additional anthropometric measurements, such as waist, hip, and neck circumferences.

### Limitations

Due to the specific site COVID-19-related restrictions, we could only collect both remote and in-person data for two of the four sites, limiting our sample size and the generalizability of the findings. We also could not conduct both types of assessment within the same session because of COVID-19 restrictions. We had to exclude 7 of 40 (18%) dyads because measurements were taken too far apart or exceeded the range of biological plausibility. In addition, our study did not collect data on whether caregiver height was measured by another adult at home or based on parent self-report. Despite its limitations, the study demonstrated the feasibility and validity of measuring height and weight in children and adults with non-research-grade equipment using videoconferencing.

## Conclusions

The findings suggest that remote weight and height measurements through videoconferencing using non-research grade equipment may be a feasible and valid approach in pediatric clinical trial studies conducted in rural communities. However, the LOAs and absolute mean difference analyses indicate that there is a need for these values to be interpreted with caution in individuals. Researchers should weigh the benefits and limitations of remote height and weight measurements and their intervention effect size when deciding if the remote measurements are suitable for their studies.

## Data Availability

The datasets used and/or analyzed during the current study are available from the Data Coordinating and Operations Center for the ECHO IDeA States Pediatric Clinical Trials Network.

## Abbreviations

BMI: Body mass index
BMIaz: Body mass index adjusted z score
SD: Standard Deviation
CI: Confidence interval
LOA: Limits of agreement
